# Similarities and differences between on-scalp and conventional in-helmet magnetoencephalography recordings

**DOI:** 10.1371/journal.pone.0178602

**Published:** 2017-07-24

**Authors:** Lau M. Andersen, Robert Oostenveld, Christoph Pfeiffer, Silvia Ruffieux, Veikko Jousmäki, Matti Hämäläinen, Justin F. Schneiderman, Daniel Lundqvist

**Affiliations:** 1 NatMEG, Department of Clinical Neuroscience, Karolinska Institutet, Stockholm, Sweden; 2 Donders Institute for Brain, Cognition and Behaviour, Radboud University, HE Nijmegen, The Netherlands; 3 Department of Microtechnology and Nanoscience—MC2, Chalmers University of Technology, Gothenburg, Sweden; 4 Department of Neuroscience and Biomedical Engineering, Aalto University, Aalto, Espoo, Finland; 5 Aalto NeuroImaging, Aalto University, Aalto, Espoo, Finland; 6 Athinoula A. Martinos Center for Biomedical Imaging, Department of Radiology, Massachusetts General Hospital/Harvard Medical School, Charlestown, MA, United States of America; 7 Harvard-MIT Division of Health Sciences and Technology, Cambridge, MA, United States of America; 8 Institute of Neuroscience and Physiology, University of Gothenburg and MedTech West, Göteborg, Sweden; Australian Research Council Centre of Excellence in Cognition and its Disorders, AUSTRALIA

## Abstract

The development of new magnetic sensor technologies that promise sensitivities approaching that of conventional MEG technology while operating at far lower operating temperatures has catalysed the growing field of on-scalp MEG. The feasibility of on-scalp MEG has been demonstrated via benchmarking of new sensor technologies performing neuromagnetic recordings in close proximity to the head surface against state-of-the-art in-helmet MEG sensor technology. However, earlier work has provided little information about how these two approaches compare, or about the reliability of observed differences. Herein, we present such a comparison, based on recordings of the N20m component of the somatosensory evoked field as elicited by electric median nerve stimulation. As expected from the proximity differences between the on-scalp and in-helmet sensors, the magnitude of the N20m activation as recorded with the on-scalp sensor was higher than that of the in-helmet sensors. The dipole pattern of the on-scalp recordings was also more spatially confined than that of the conventional recordings.

Our results furthermore revealed unexpected temporal differences in the peak of the N20m component. An analysis protocol was therefore developed for assessing the reliability of this observed difference. We used this protocol to examine our findings in terms of differences in sensor sensitivity between the two types of MEG recordings. The measurements and subsequent analysis raised attention to the fact that great care has to be taken in measuring the field close to the zero-line crossing of the dipolar field, since it is heavily dependent on the orientation of sensors. Taken together, our findings provide reliable evidence that on-scalp and in-helmet sensors measure neural sources in mostly similar ways.

## Introduction

The introduction of superconducting whole-head magnetoencephalography (MEG) recording systems [1] was a significant step forward for non-invasive recordings of neural activity in the human brain. As in electroencephalography (EEG), the MEG signal reflects mainly the integrated currents stemming from post-synaptic potentials [2]. In comparison to EEG, MEG offers much more precise estimates of the spatial location of sources [2–4]. A remaining technical limitation of whole-head MEG systems, however, is that the sensors (magnetometers and/or gradiometers) are at fixed locations inside a helmet-shaped dewar. Thermal insulation is required to keep the MEG sensors in a superconducting state [5,6]. Furthermore, the helmet has a fixed size that allows recordings of subjects with a head circumference of up to ~61 cm (average head size for males: 57 cm, females: 55 cm [7]). This design typically leaves additional space between parts of a subject’s scalp and the inside of the helmet. Together, these factors result in measurements with conventional in-helmet MEG sensor arrays with a typical distance of 20–40 mm between the sensors and the subject's scalp.

To obtain spatially detailed measurements of the underlying neural activity, MEG sensors should be as close to the brain as possible. Hence, positioning MEG sensors directly on the scalp would be ideal. While EEG sensors are indeed placed directly on the scalp, the skull and scalp have very different conductivities and it has proved challenging to take these factors into account in source modelling [8,9]. MEG sensors are however not troubled by these factors; the skull and the scalp are transparent to magnetic fields allowing a more straightforward solution for the source estimation problem with MEG.

With emerging sensor technologies, on-scalp MEG measurements have become possible. Two prominent and promising sensor technologies exist, high-critical-temperature (high-*T*_c_) superconducting quantum interference devices (SQUIDs) [10–12] and optically pumped magnetometers (OPMs) [13,14]. High-*T*_c_ sensors are superconducting at much higher temperatures (<90 K; 77 K is the typical operation temperature with liquid nitrogen cooling) than conventional low-*T*_c_ sensors (< 9 K; 4 K is the typical operation temperature with liquid helium cooling). Insulation requirements are thus less severe for high-*T*_c_ sensors, and, consequently, the sensor-to-room distance can be reduced from 20–40 mm to less than 1 mm, at least for single sensors [11]. For multi-channel arrays, however, it may not be feasible to achieve such a short stand-off for all of the on-scalp sensors. However, even single-channel OPM systems cannot get as close to the scalp due to the thermal shielding they employ for the hot gas. OPMs currently allow for a minimum stand-off of ~4 mm [15]. Regardless, the improved sensor-to-cortex proximity that these new sensor technologies allow in comparison to conventional MEG sensor technology may provide new information about the neural activity of the brain. Simulations have indicated on-scalp MEG promises improved spatial precision in estimating the location of neural sources and better separation of source activations [16,17].

In this study, we explore how differences in sensor-to-cortex proximity between high-*T*_c_ and conventional low-*T*_c_ MEG sensors influence the measurements of a cortical source. We hence compared the results from measurements using a single high-*T*_c_ magnetometer (hereafter called “on-scalp MEG”) to measurements from conventional low-*T*_c_ MEG magnetometers (hereafter called “in-helmet MEG”). For this comparison, we used electric median nerve stimulation to generate somatosensory evoked fields (SEFs) originating from the primary somatosensory cortex. We focused on the early and well-defined N20m component of the SEF, a source that is well-modelled with a single current dipole located at the hand area of the primary somatosensory (S1) cortex [18,19]. The N20m is consistently found in all healthy subjects and is also known to be a component that is robust with regard to habituation [20,21], thereby allowing for large numbers of trials across repeated measurements to be compared directly.

Due to the differences in proximity to the cortex between these recordings, we expected an N20m peak of higher amplitude for the on-scalp MEG compared to in-helmet MEG measurements, simply because on-scalp sensors are closer to the source. Getting closer to the source should, however, not change the timing of the evoked response, so we expected a very similar *temporal* profile of the N20m component between measurements.

## Section 1: Neuromagnetic recordings

### Materials and methods

#### Subject

The involvement of human volunteers was performed in accordance with the technical development prerogative of the Swedish law for ethical approval of research. The subject orally consented to participating in the study.

#### Equipment

All measurements were carried out in a two-layer magnetically shielded room (MSR; model AK3b from Vacuumschmelze GmbH & Co. KG, Hanau, Germany) at the NatMEG facility, Karolinska Institutet, Stockholm, Sweden (www.natmeg.se).

#### In-helmet MEG

We used an Elekta Neuromag TRIUX (Elekta Oy, Helsinki, Finland) system to represent conventional MEG sensors as it is globally the predominant MEG system in use. The Elekta Neuromag TRIUX contains 102 sensor chips, each with a magnetometer channel with a pickup loop size of 21 mm × 21 mm, and two orthogonal planar gradiometer channels.

#### On-scalp MEG

We used a single-channel magnetometer with a 9 mm × 9 mm pickup loop fabricated at the Chalmers University of Technology as the on-scalp sensor. The sensor was housed inside a non-metallic cryostat (ILK Dresden), the tail of which has a diameter of 50 mm, placed at a ~2 mm distance to the subject's scalp during measurements. On-scalp MEG was sampled with a National Instruments analogue-to-digital converter connected to a laptop. More experimental details for MEG recordings with our high-*T*_c_ SQUIDs can be found in Öisjöen et al. [11] and Xie et al. [22]. We concurrently sampled the on-scalp MEG using the Elekta Neuromag TRIUX system. The output of the SQUID electronics was fed into one of the analogue miscellaneous (MISC) channels of the Elekta system that allowed us to sample the on-scalp MEG data with the same clock as the in-helmet MEG data.

#### Physiological data

Electrooculography (EOG), and electrocardiography (ECG) electrodes were attached to the subject and recorded with the Elekta system. Ten head position indicator (HPI) coils were then positioned on the subject's head, evenly distributed across the 128 cap slits of a custom montage EEG cap (EasyCap) placed on the subject’s head. Four scalp electrodes were added to measure EEG with two active channels. These were positioned orthogonally to the projected N20m maxima to measure the sensory evoked field.

#### Experimental procedure

A central part of the preparation was to estimate the scalp locations of the positive and negative extrema of the dipolar N20m pattern such that optimal measurement points could be planned for the single-sensor on-scalp recordings on the next day. Through all recordings, we used left median nerve stimulation at the wrist to generate SEFs. A minimum of 1,000 stimulations per recording was delivered over the course of at least 5 minutes with a repetition rate of 2.8 Hz. Stimulus intensity was 6.0 mA in pulses of 200 μs that induced a slight movement of the thumb. Vacuum pillows were used to increase the subject's comfort and reduce movement of the head and body. The subject (one of the authors), was highly motivated and not naïve to the purpose of the experiment.

An overview of the experimental procedure is presented in Table 1. On day one, we performed a whole-head in-helmet MEG recording of the subject. Dipoles were fitted for each sampling point around the N20m for this recording (15.0 ms– 25.0 ms; 51 samples, 0.2 ms apart). The fitted dipole at the latency with the least residual variance was chosen to be the single active source in a spherical forward model based on the anatomy of the subject acquired through T1-weighted magnetic resonance images. This forward model thus provided us with estimates of where the positive and negative N20m extrema would be on the scalp surface.

**Table 1:** Overview of the procedure for the recordings.

*Day one procedure:*
1. A SEF recording was made using conventional whole-head in-helmet MEG.
2. Equivalent current dipoles (ECDs) were fitted to the N20m component of the SEF for each sample from 15.0 to 25.0 ms.
3. The source pattern of the dipole with the least residual variance was projected onto the scalp using a volume conduction model based on the subject's anatomy.
4. Based on the projected on-scalp dipole source pattern, ten measurement sites were marked along the curve connecting the centres of the two polar extrema
*Day two procedure:*
5. An additional SEF recording was made using conventional whole-head in-helmet MEG; hereafter referred to as the “before” recording.
6. Based on the prepared EEG-cap from day one, on-scalp MEG recordings for each of the ten planned measurement sites were conducted.
7. A final SEF recording was made using conventional whole-head in-helmet MEG; hereafter referred to as the “after” recording.

The positions of the extrema corresponded roughly with electrode positions of the 128-channel EEG cap. Ten on-scalp recording positions were selected and printed out on laminated paper. This printout would serve as a guide for the handler of the cryostat for on-scalp recordings on the following day. The guide was attached to the EEG cap on the subject’s head by using the electrode positions corresponding to the predicted extrema for the N20m as reference points. The placement of the EEG cap was photographically documented, and the distance from the nasion to the bottom of the cap was measured such that the EEG-cap with the guide attached could be placed in an identical position on the day of the on-scalp recording. The recording positions were digitized with a Polhemus Fastrak tracker. To minimize digitization error for the experimenter handling the Polhemus stylus, the laminated layout was printed with small holes fitting the tip of the stylus.

On day two, the cryostat was aligned with respect to the markers on the guide by hand-adjusting a wooden articulated armature that supported the cryostat above the subject's head inside the MSR. Alignment with respect to position was achieved via alignment of markers on the cryostat with those on the subject's head. Alignment with respect to angle was achieved via manually tilting the cryostat such that the semi-conical gap between the planar cryostat lid and the curved head surface was as even as possible for at least three pairs of diametrically opposing points on the cryostat lid. The armature was then locked into place for each recording position with an estimated accuracy in position of ± 4 mm and an estimated accuracy in angle (with respect to the normal of the head surface) of ± 5 degrees (verified with a head-phantom). An overview of the experimental procedure can be seen in Table 1.

**Fig 1:**
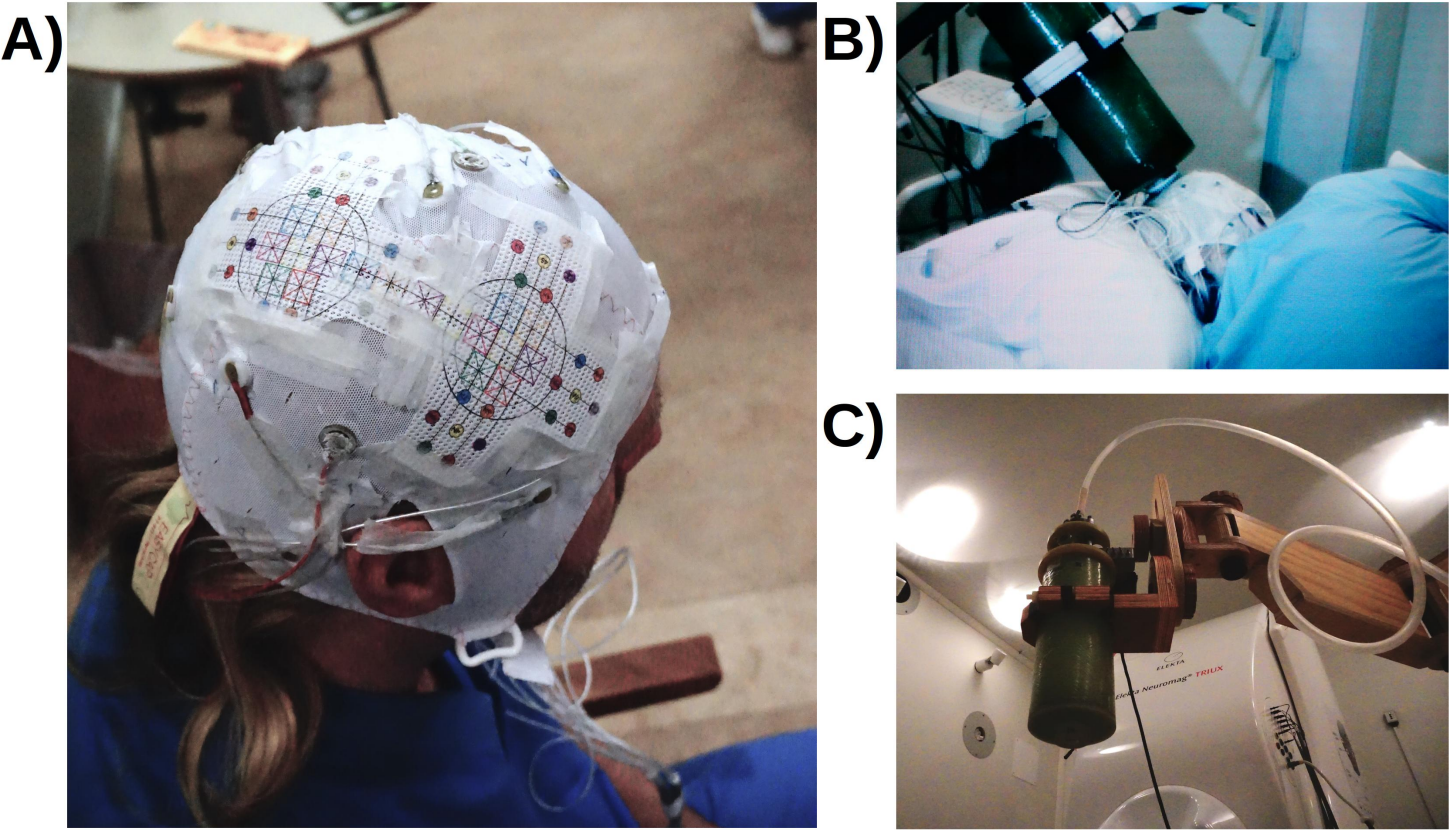
Photographs of experimental procedure. **A)** The subject wearing the EEG-cap with the laminated guide. **B)** The subject lying on the recording bed with the cryostat in position. **C)** The cryostat on the wooden articulated armature.

In total, fourteen recordings were performed on day two. First, an empty room recording was obtained followed by a whole-head in-helmet recording that preceded the ten on-scalp recordings. During the on-scalp recordings, data were also acquired from the 102 magnetometers in the whole-head system to generate an estimate of the noise level within the room. Between each recording, the stability of the subjective sensation of the electrical stimulation was verified, and the subject’s alertness was assessed with the Karolinska Scale [23]. After the on-scalp recordings, a whole-head in-helmet recording was repeated to validate the stability of the field topography of the N20m. We concluded with an additional empty room recording. The cap with the guide and the cryostat in a recording position can be seen in Fig 1.

#### Acquisition of MEG data

The signal from the high-*T*_c_ SQUID was acquired through two different systems simultaneously, the electronics analogue-to-digital converter and one of the MISC channels of the Elekta system. Only data acquired with the Elekta system are discussed below. All data were sampled at 5,000 Hz, low-pass filtered at 1,650 Hz and high-pass filtered at 0.1 Hz.

#### Pre-processing of data

The open-source package FieldTrip was used for all analyses [24]. Recorded data was first low-pass filtered at 300 Hz and line-filtered at 50 Hz including all 50 Hz harmonics up to the low-pass filter. The raw data was then cut into epochs of 300 ms, spanning from 100 ms pre- to 200 ms post-stimulation. All epochs were de-meaned by using the mean activity of the pre-stimulation period. The epoched on-scalp data was cleaned automatically first by removing all epochs in which the EOG channel had values greater than 250 μV and was subsequently manually cleaned by removing epochs with large variance. At least 1,000 epochs remained for further analysis.

#### Volume conduction and forward models for MEG data

A local spherical volume conductor model was created based on a T1 magnetic resonance image of the subject. The image was co-registered to the subject's head shape with 426 digitization points distributed over the head surface from the Polhemus Fastrak. Subsequently, the image was segmented into brain, skull, and scalp tissues. Based on the brain tissue, a single shell spherical model was created [25]. A forward model for dipole fitting was created based on the volume conductor model and the known geometry of the whole-head sensor array.

#### Estimating the in-helmet and on-scalp MEG topographies

We modelled the N20m activation with a moving, single dipole, the strength of which varied with time. The position and time course of this dipole were estimated based on the “before” and “after” whole-head recordings for each of the time samples from 15 ms to 25 ms in steps of 0.2 ms. Based on the fitted dipole, a second forward model was created with a single active source at the location of the peak of the fitted dipole. This forward model was based on the “after” recording. For the on-scalp topography, it was projected to 10,000 points on the scalp surface of the subject, whereas it was projected onto the 102 magnetometer locations for the in-helmet topography.

To summarize, for each of the ten scalp positions and both the “before” and “after” in-helmet recordings, we have a *predicted value* and a *measured value* of the time course of the neuromagnetic field. For example, the *predicted value* for the “before” recording is based on forward calculating the neuromagnetic field emanating from a moving dipole whose time-dependent activation strength is estimated from the “before” in-helmet recording. The *measured* value is the “before” recording itself. Similarly, we have a *predicted value* and a *measured value* of the time course of the neuromagnetic field for the on-scalp recording. In this case, the *predicted value* is based on the “after” in-helmet data. For all *measured values*, we used a bootstrapping procedure to estimate 95% confidence intervals.

### Results

#### In-helmet MEG

**Fig 2:**
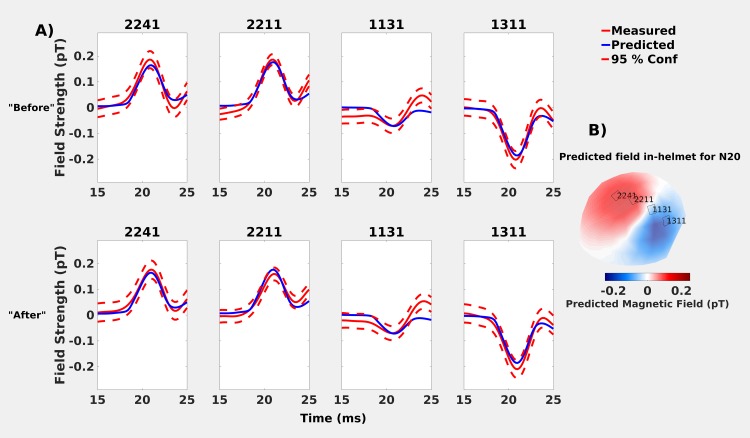
Comparisons of predicted (blue solid lines) and measured (red solid lines) neuromagnetic field strengths for the in-helmet recordings in the “before” and “after” datasets. Predicted values are based on a moving dipole fitted to the “after” whole-head recordings, measured values are presented with 95% confidence intervals (red dashed lines). **A)** *Top row*: “Before” recording: the values overlap and the N20m activation peak occurs at 20.8 ms. *Bottom-row*: “After” recording: predicted and measured values overlap again, but the N20m peak occurs slightly later (21.0 ms). **B)** Predicted in-helmet field topography and selected sensor positions.

The predicted and measured values for the in-helmet recordings showed very similar time courses for all sensors over the line intersecting the polar extrema (Fig 2A). Note the peak of the N20m is delayed (0.2 ms later) in the “after” recording as compared to the “before” one.

The (minimum, median, and maximum) distances between the subject's scalp and the sensors were (1.9, 2.9, and 3.9) cm and (1.8, 3.0, and 4.3) cm, for the “before” and “after” in-helmet recordings, respectively. This is within the typical range (as outlined above in the introduction). Four magnetometers, connecting the two extrema of the predicted in-helmet field topography, were chosen to compare with the predicted values (sensors 2241, 2211, 1131 and 1311; Fig 2B).

#### On-scalp MEG

**Fig 3:**
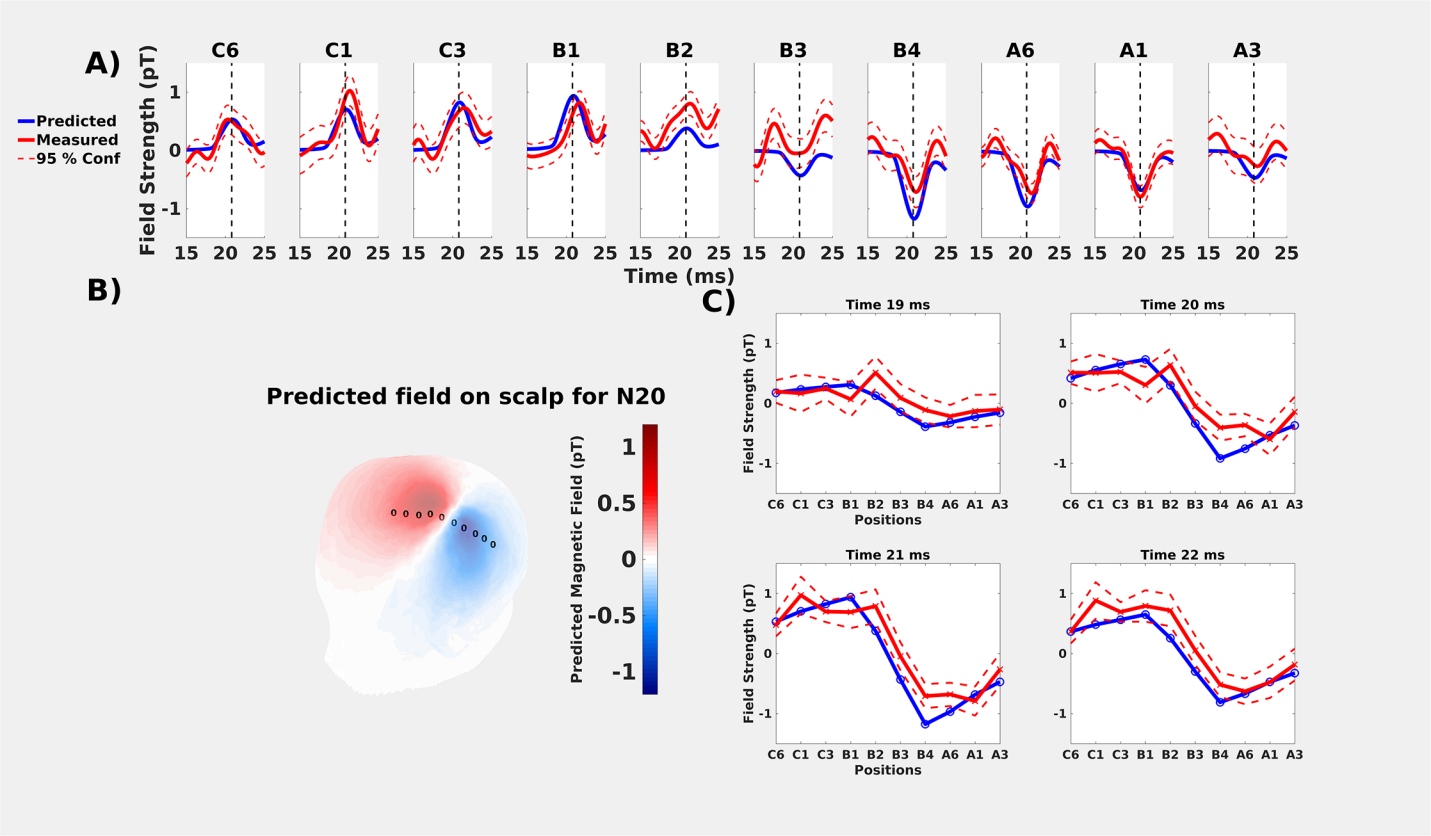
Comparisons of predicted (solid blue lines) and measured (solid red lines) values for on-scalp recordings. All predicted values are based on a moving dipole fitted to the whole-head “after” recording that was performed immediately after the ten on-scalp positions. Measured values include 95% confidence intervals (red dashed lines). **A)** On-scalp comparisons for all ten recording positions. The vertical dashed line is the estimated peak of 21.0 ms. **B)** predicted on-scalp topography of the neuromagnetic field for the time point with the lowest residual variance (20.8 ms). Measurement points are marked with circles. From left to right they are: C6, C1, C3, B1, B2, B3, B4, A6, A1 and A3 (same order as in A)) **C)** Spatial distribution of the predicted and measured values at specific time points.

Overall, the predicted and measured on-scalp field strengths showed very similar development over time across all 10 measurement sites (Fig 3A). The greatest discrepancies in field amplitude were found near the zero crossing (positions B2-B3; Fig 3A). Furthermore, the latencies of the measured peaks in the N20m activations were slightly later or earlier than predicted (Fig 3 & Table 2). These temporal discrepancies were up to 0.6 ms, greater than the 0.2 ms discrepancies found between the “before” and “after” in-helmet recordings.

**Table 2:** Discrepancies in timing between predicted and measured latencies for the peak N20m activation for the on-scalp recordings.

	Predicted	C6	C1	C3	B1	B2	B3	B4	A6	A1	A3
Timing (ms)	21.0	20.4	21.4	21.6	21.6	21.4	20.4	21.2	21.4	20.8	21.2
Difference	-	-0.6	+0.4	+0.6	+0.6	+0.4	-0.6	+0.2	+0.4	-0.2	+0.2

### Discussion

As expected, the predicted neuromagnetic field topography for the on-scalp (Fig 3B) recording is more spatially compact than for the in-helmet (Fig 2B). This is because of the differences in sensor-to-cortex proximity between on-scalp and in-helmet MEG sensors. Our results thus show that the N20m amplitude as measured from a series of single-channel on-scalp recordings can mostly be accurately predicted by the combination of a dipole model based on a whole-head MEG recording and a volume conduction model. The results, however, also show that the similarity of the predicted and measured signals was reduced for measurement points that were close to the dipole field zero-line crossing (cf., results for recoding positions B2 and B3 in Fig 3).

Finally, we found unexpected temporal differences between the predicted and measured N20m peaks (Table 2). These differences had no clear pattern with respect to measurement position.

The comparisons between in-helmet and on-scalp recordings depend on the quality of several factors, such as the reliability of initial measurements, accuracy in dipole source projection and measurement planning, accuracy in actual measurements, as well as reliability and quality in sensory stimulations between measurements. We therefore explored and assessed the validity and reliability of these factors in the following Section 2.

## Section 2: Assessing validity and reliability

The comparison between predicted and measured values for the neuromagnetic fields at the scalp resulted in both temporal and spatial differences. To explore the cause of the differences, we conducted several exploratory analyses focusing on the reliability of the results.

### Overview of exploratory analyses

1. The differences in the N20m amplitude might be due to sensory *habituation*, i.e. changes in cortical response over time due to the repeated stimulation. Therefore, we assessed whether the predicted amplitude changed significantly from the “before” recording to the “after” recording.
2. The differences in the N20m amplitude might be due to different sources being active due to the *stimulator repositioning* that was unavoidable throughout the experiment. Therefore, we assessed whether the N20m would change significantly as a consequence of repositioning the median nerve stimulator.
3. The differences in peak latencies and N20m amplitudes might be due to *the head and dipole modelling choices*. Therefore, we assessed how different modelling choices changed the predicted time courses.
4. The differences in peak latencies might be due to *sensitivity differences* between the on-scalp and in-helmet recordings. We therefore assessed the difference in sensitivities the in-helmet and on-scalp sensors have to different parts of the brain.
5. The differences in the N20m amplitude might be due to the uncertainty related to the *orientation* of the coil of the on-scalp sensor. Therefore, we assessed whether differences in amplitude could be related to the unknown orientation of the coil.

### (1) Habituation to median nerve stimulation

#### Purpose

During these analyses, we assessed if there were any potential habituation to median nerve stimulation between the “before” and “after” in-helmet recordings (Section 1). Habituation would have the effect that the cortical responses would reduce in amplitude over time and might thus explain some of the observed differences in amplitude.

#### Methods

We assessed the potential habituation effect by comparing the predicted on-scalp fields for fitted dipoles from the “before” and “after” recordings. If there were any effects of habituation, the predicted field for the “after” recording should be lower in amplitude than the one from the “before” recording.

The head model used for predicting the fields was the same as in Section 1, but with the modelled source location being determined by the dipole fits from the “before” and “after” recordings respectively.

#### Results and discussion

**Fig 4:**
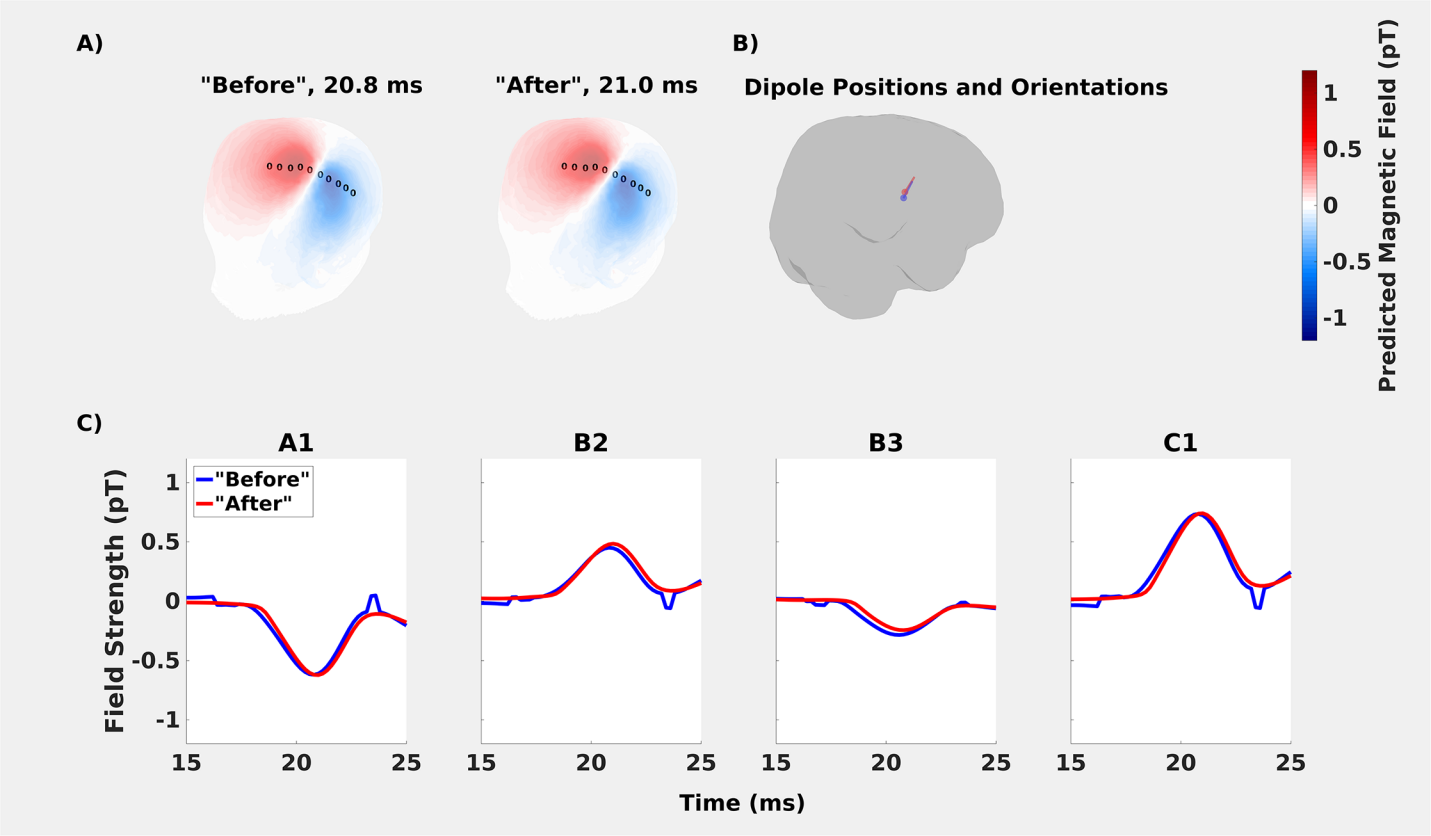
Comparing the “before” and “after” recordings to assess habituation effects. **A:** Topographies based on the fitted dipoles were very similar. Measurement points are marked with circles. From left to right they are. C6, C1, C3, B1, B2, B3, B4, A6, A1 and A3 (same order as in A) **B:** The fitted dipoles shown on cortex. **C:** Only minor differences were found in peak amplitudes. The peak for the “after” recording occurred 0.2 ms after the “before” recording.

In comparing the “before” and “after” in-helmet recordings, no major differences were found between estimated scalp topographies, dipole positions, or dipole orientations. The distance between dipoles in the “before” and “after” recordings was 4.5 mm. With the spatial resolution for MEG source reconstructions being on the order of 3–15 mm [2,26,27], this is a negligible difference. The angle between “before” and “after” dipole moments was 7.7° (Fig 4). Differences in peak amplitude between the “before” and “after” recordings were also negligible (Table 3). For time points before and after the dipolar peak, the dipole model did not fit a smooth curve in the case of the “before” recording. We interpret this an uninteresting feature of fitting noise (i.e., time points outside the window of the N20m activation) with a dipole, the solution of which is not well determined.

**Table 3:** Differences in scalp peak amplitude at the ten measurement positions, prediction based on the “before” and “after” recordings.

Peak Amplitude	C6	C1	C3	B1	B2	B3	B4	A6	A1	A3
“Before” (pT)	+0.54	+0.73	+0.89	+0.91	+0.45	-0.28	-0.95	-0.84	-0.62	-0.43
“After” (pT)	+0.53	+0.73	+0.88	+0.94	+0.48	-0.24	-0.90	-0.81	-0.61	-0.42
Difference (pT)	0.01	0.00	0.01	0.03	0.03	0.04	0.05	0.03	0.01	0.01

#### Conclusion

The *habituation* results show that the dipole fits based on “before” and “after” in-helmet recordings are not significantly different from one another. This confirms the conjecture that N20m activations are not strongly effected by habituation [20,21]. Thus, the spatial and temporal differences between the predicted and measured on-scalp fields cannot be readily explained by habituation.

### (2) Stimulator repositioning

#### Purpose

Movement of the stimulator may have occurred as the subject had to reposition himself between some of the on-scalp recordings. However, care was taken to attach the stimulator to the same place and thus elicit the same movement of the thumb. To assess whether there were effects on N20m amplitudes and latencies from repositioning the stimulator, and thereby potentially activating different cortical sources, we performed five additional whole-head recordings in a separate session after the benchmarking measurements. These experiments were carried out on the same subject, with the same stimulator, and recorded with the same whole-head MEG system and settings as in the benchmarking measurements presented in Section 1, above.

#### Methods

During these measurements, we removed and replaced the stimulator on the subject’s wrist between each of the five recordings to emulate the repositioning done during the repeated on-scalp recordings. This allowed us to assess what effect repositioning might have on the fitted dipoles. As during the on-scalp recordings, care was taken to reposition the stimulator on the same spot so that it would result in a stimulation as similar as possible to the one before. All the analysis steps were identical to the ones applied in the aforementioned “before” and “after” whole-head recordings in Section 1.

#### Results and discussion

**Fig 5:**
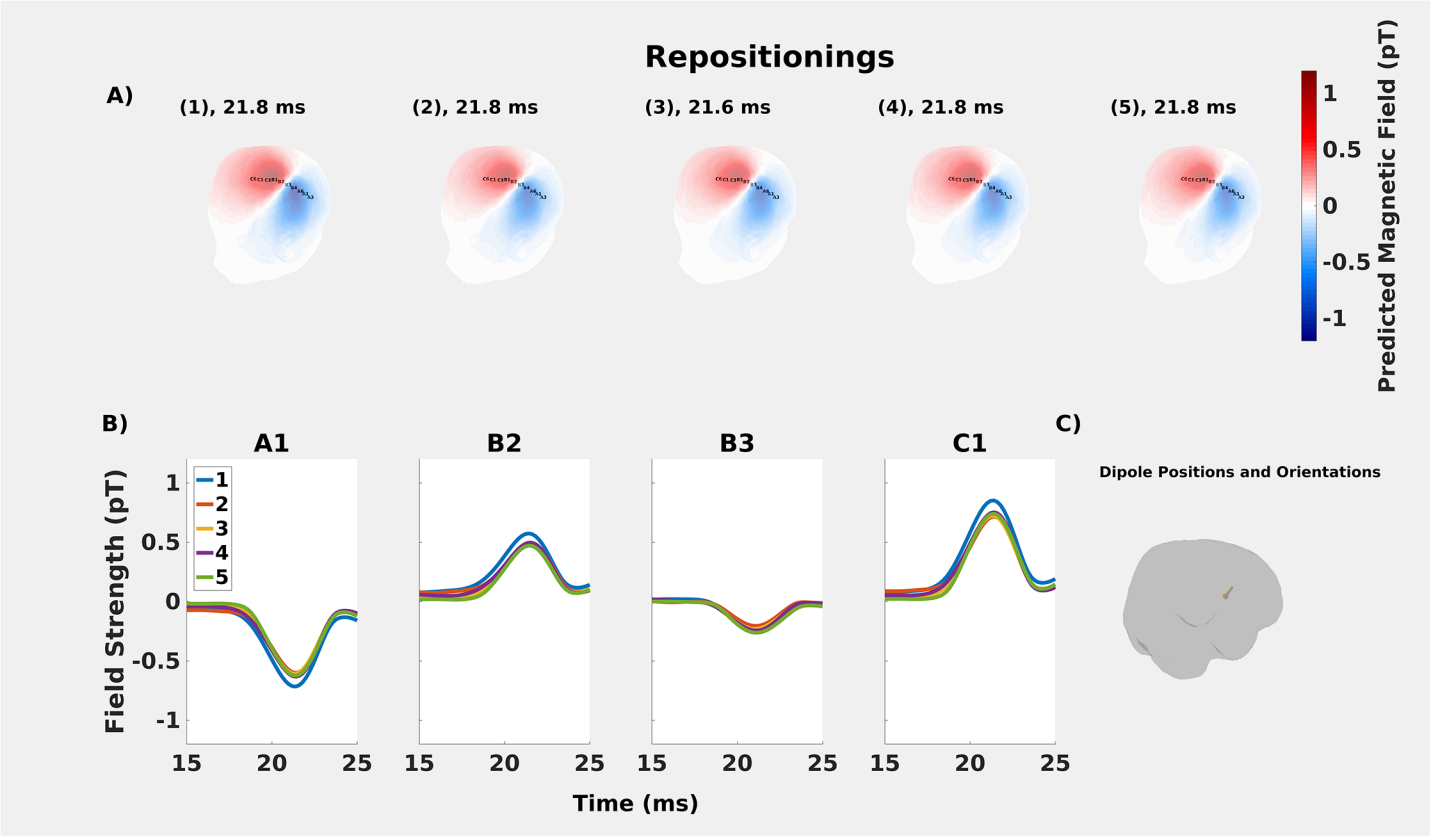
Repositioning analyses. **A:** Comparisons of predicted on-scalp topographies for the whole-head recordings for the repositioning (1–5). **B:** The estimated dipoles are temporally stable across the repositionings. **C:** The maximum distance between estimated dipoles was maximally ~2 mm across the repositionings.

The resulting differences in positions of the fitted dipoles were within ~2 mm and on average differed 1.4 mm (Table 4). Angles between the moments of the fitted dipoles were within ~7° (Table 5). Temporal reliability was also high: only one fitted dipole had a deviation in peak timing of 0.2 ms before the others (Fig 5). Compared to the peak time discrepancies found in the on-scalp measurement (Fig 3), this discrepancy is smaller by a factor of three. Repositioning was thus found to activate a consistent source, the variation in which does not account for the peak time discrepancies reported in Section 1.

**Table 4:** Distances between estimated positions of fitted dipoles for recordings where the stimulator was repositioned.

Distance (mm)	1	2	3	4	5
**1**	0	1.9	1.9	2.2	1.7
**2**		0	0.4	1.3	1.0
**3**			0	1.6	1.1
**4**				0	0.8
**5**					0

**Table 5:** Angles between estimated moments of fitted dipoles for recordings in which the stimulator was repositioned.

Angle (°)	1	2	3	4	5
**1**	0	2.6	1.4	1.7	4.5
**2**		0	3.6	3.5	6.8
**3**			0	0.7	3.2
**4**				0	3.4
**5**					0

#### Conclusion

The *repositioning* results show that the discrepancies between predicted and measured magnetic fields for the on-scalp recordings are of a different order than what is induced in the in-helmet recording by repositioning the median nerve electrodes. The spatial and temporal differences between the predicted and measured on-scalp fields cannot therefore readily be explained by the repositioning of the median nerve electrode during the on-scalp recordings. To be precise, the angles between dipole moments and distances between dipole positions were less than 7° and 2 mm, respectively.

### (3) Volume conduction models and dipole fitting options

#### Purpose

Given the consistency of the N20m response and its dipolar presentation, we investigated how dependent it was on fitting options and the volume conduction model used.

#### Methods

The prediction of the on-scalp magnetic field is dependent on a number of factors. We tested three modelling factors across twelve different modelling choices that are understood to have strong effects on the predictions. The factors we assessed were: *Volume Conductor* (Single Sphere; Single Shell [28–30]), *Sensors included in the dipole fit* (Magnetometers; Gradiometers; Magnetometers *and* Gradiometers) and *Rank of the data* (Full Rank; Reduced Rank (removing the weakest orientation)).

#### Results and discussion

**Fig 6:**
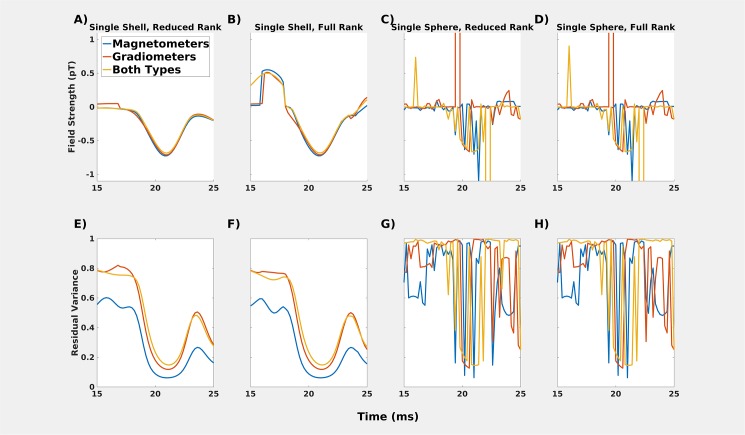
**A-D: Comparisons of different head models for the “after” recording for position A1.** Single sphere models (**C** & **D**) do not result in a smoothly-varying dipole field pattern. Single shell models, however (**A** & **B**) show very similar estimations of the magnetic field generated by the source underlying N20m. The full rank model is less stable than the reduced rank model, which also shows some temporal discontinuities, e.g., before the peak of the N20m activation. **E-H:** Residual variance for the models.

The choice of volume conductor model made a significant difference in estimates of the on-scalp field, with single sphere volume conductors being very unstable. The stability of the single shell volume conductor depended to some degree on the rank of the data. The full rank model was less stable (Fig 6B) around the N20m peak compared to the reduced rank model (Fig 6A). The instability of these full rank fits for the single shell volume conductor presumably depend on the fitting of noise before and after the dipolar field. For the single sphere volume conductors, the fit is bad throughout.

#### Conclusion

The *modelling* results show that the most stable on-scalp estimates are obtained with a single shell volume conductor and reduced-rank data. Whether only magnetometers, only gradiometers, or both sensor types are included in the dipole fit does not have a significant impact (the lower residual variance for magnetometers is probably due to fewer data points having to be fitted). We used a single shell volume conductor with reduced rank of the data with both magnetometer data and gradiometer data throughout Section 1. As such, the differences in predicted and measured on-scalp fields cannot readily be explained by the modelling choices we made for projecting the estimated dipolar field to the scalp.

### (4) Sensitivity differences between sensor types

#### Purpose

In this analysis, we investigated whether the differences in timing between predicted and measured time courses could be explained in differences between sensitivity profiles between the on-scalp and in-helmet [31].

#### Methods

**Fig 7:**
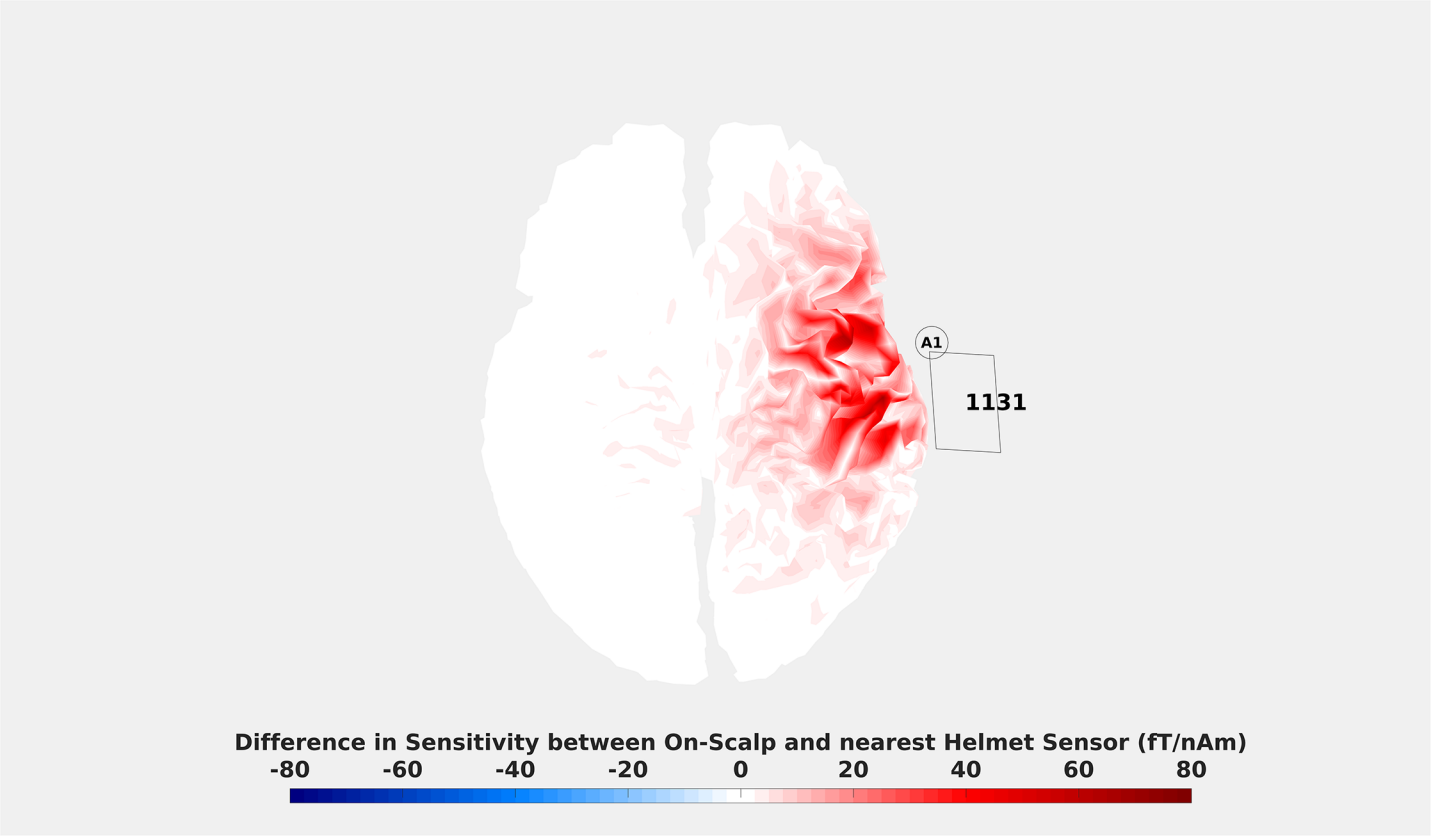
Difference in sensitivity between position A1 and helmet sensor MEG1311. The colouring indicates the difference in field magnitude that a source on the cortex with a current of 1 nAm would generate for the on-scalp and in-helmet sensors. Unsurprisingly, it can be seen that the on-scalp sensor is more sensitive than the in-helmet sensor for virtually every source over the target region. The magnitude of that difference, however, is manifested in a spatially heterogeneous manner. Note that similar maps showcasing spatial heterogeneity can be made between any position and any helmet sensor.

For this purpose, we created a map of the cortex highlighting differences in sensitivity between the on-scalp and in-helmet recordings (Fig 7).

#### Results and discussion

The results show that the sensitivity is generally higher for on-scalp sensors. This finding is somewhat trivial as it reflects the decrease in stand-off on-scalp sensors enable in relation to in-helmet ones. The results also show that the sensitivity differences are distributed in a spatially heterogeneous manner. This finding may explain the observed differences in timing of the peak N20m activations between predicted and measured on-scalp fields. The source activity that gives rise to N20m propagates over the cortex, meaning that differences in sensitivity over the scalp can affect how the predicted and measured on-scalp activities match.

#### Conclusion

The *sensitivity* results show that the temporal differences between predicted and measured magnetic fields for on-scalp recordings may be explained by the spatial variation of the sensitivity to cortical sources.

### (5) Sensor orientation during on-scalp recordings

#### Purpose

The neuromagnetic field to which MEG is sensitive is primarily generated by cortical currents [32]. Magnetic fields are, in general, vector fields: thus they contain both a magnitude and orientation. The orientation can be decomposed in radial and tangential components. MEG systems are designed to record the radial component of the magnetic field as the tangential components include the contribution of volume currents that are secondary to primary (source) currents. In our recordings, an attempt was made to place the on-scalp sensor such that it would record the radial component of the MEG field. During the experimental procedure, we therefore oriented the tail of the sensor flush with the head surface. The diameter of the cryostat lid is 50 mm. Since the head surface is curved, there is uncertainty in the orientation of the sensor that translates into an uncertainty of the MEG signals.

#### Methods

To estimate this uncertainty for the orientation of the on-scalp sensor (at the centre of the cryostat tail), we estimated the MEG signal for the full range of orientations that the cryostat tail could achieve, with it still being flush with the scalp. We defined this range as the *orientation* confidence interval, which included all points on the scalp surface not further than 25 mm away from the centre of the cryostat tail. The magnitude of the magnetic field is maximal close to the zero-line crossing, directly above the source. However, it is here where the radial component of the neuromagnetic field is minimal. Magnetometers that are flush with the head surface should therefore pick up no magnetic flux. If, however, the sensor is not flush with the head surface, then it will be sensitive to the tangential component of the field.

#### Results and discussion

**Fig 8:**
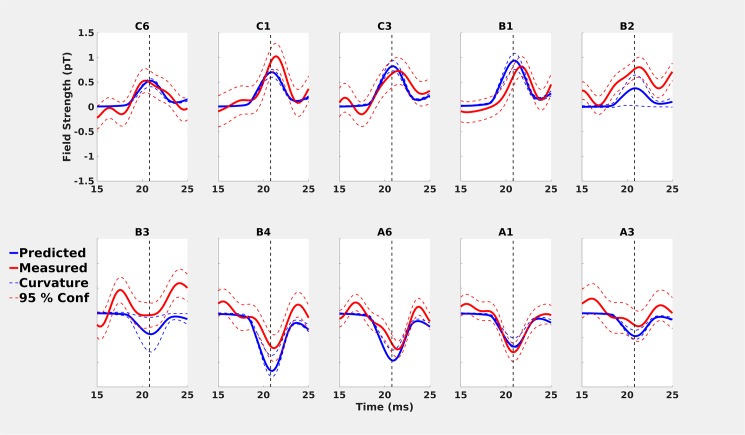
Comparisons of predicted (solid blue lines) and measured (solid red lines) values for on-scalp recordings with confidence intervals estimated by changing the orientation of the on-scalp sensor. The orientation bounds are based on the maximal uncertainty due to the curvature of the head and the size of the cryostat lid.

We found that the widths of these orientation confidence intervals were greatest around the zero-line crossing (B-positions; Fig 8). This indicates the uncertainty of the sensor orientation influenced the uncertainty of the MEG signal. It follows that the uncertainty of the orientation of the sensor should most greatly translate into uncertainty of the MEG signal where the tangential component of the magnetic field is maximal i.e., at the zero-line crossing.

#### Conclusion

The *orientation* results show that the uncertainty of the orientation of the sensor translates into the greatest uncertainty closest to the source–that is at the zero-line crossing. The amplitude differences at the zero-line crossing (Fig 8, B-positions) may thus be affected by the uncertainty regarding the orientation of the sensor.

### Discussion

The exploratory analyses run to assess the validity and reliability of the findings reported in Section 1 indicate that the temporal differences found in peaking times between predicted and measured fields may be explained by the spatially heterogeneous differences in *sensitivity* between on-scalp and in-helmet recordings.

For the spatial differences, the *orientation* effects indicate that the differences found close to the zero-line crossing may be explained by unintentional sensitivity to the tangential components of the neuromagnetic field. The effects of *habituation*, *stimulator repositioning*, and *head modelling choices* could not independently explain the differences between predicted and measured fields in any significant manner.

## General discussion

The main challenge for comparing sensors in MEG is to accurately identify the source of any differences found between compared systems. Are they due to differences in how the sensors detect the signal coming from the brain or are there other unintentional confounds?

### Similarities and differences between on-scalp and in-helmet recordings

We have provided evidence that the on-scalp recordings resulted in measurements that are mostly in line with the predictions that can be made based on in-helmet data. Differences in amplitude of the signal fell within the predicted confidence intervals (Figs 2 & 7). Differences in the temporal evolution of the N20m component may be explained by differences in sensitivity between the two types of recordings (Fig 7). In single dipole modelling, it is assumed that components originate from a point source, but the reality is rather that they originate from patches of cortex over which cortical activity propagates. The differences in peak timings for the N20m component is indicative of differing levels of sensitivity to these patches of cortex between the sensor types. This suggests that a distributed model of the source activity is more appropriate [33,34]. However, in standard distributed models, one assumes between one and ten thousand sources per hemisphere [2,27,35,36]. Distributed models are therefore underdetermined and thus require *a priori* assumptions, such as L1 or L2 norms [2,37]). Further parameters need to be considered, such as depth weighting, cortical constraints on sources and noise models. Full-head MEG recordings with hundreds of sensors furthermore require regularization of the solution to avoid fitting the noise [38]. The model assumptions and the regularization parameter have a significant impact on source estimates, and hence would affect the forward model that we use to compare the in-helmet and on-scalp data. To eliminate these effects we opted for the simplest model that we consider appropriate. While the N20m is presumably not generated by a single dipolar source, the single dipole is still a very good model of the underlying neural source [18,19,39] and performs well in comparing predicted on-scalp fields with actual measurements.

### Limitations

Our study was limited by only having a single sensor that needed to be relocated over several recordings. For example, it was only possible to study very robust and non-habituating neural sources, effectively early sensory components. Late sensory and cognitive components have more complicated source configurations than the N20m and are thus more likely to be affected by habituation. With a single sensor setup, all source analyses will have a high degree of uncertainty related to them. Among other things, one has to assume that noise conditions are roughly equivalent across recordings and that the signal of interest is stable in time.

Our results suggest the existence of important differences in sensitivity over the cortex between on-scalp and in-helmet recordings (Fig 7). In the future, it will be of interest to perform source reconstructions on recordings like these in order to detect whether the shorter distance to the brain results in the ability to separate sources that are in close proximity to one another. This necessitates multi-channel on-scalp sensor arrays.

### Future outlooks

The feasibility of on-scalp MEG with high-*T*_c_ SQUIDs that we have demonstrated here points to a future with many new possibilities of neuroscientific interest. To fully realize the neuroscientific potential, it is necessary to develop multi-sensor arrays that can acquire MEG data from the whole head simultaneously. Such developments are ongoing [11,40]. To fully exploit multi-sensor arrays, it is necessary to be able to accurately assess the locations and orientations of sensors because the tangential field above the zero-line crossing (Fig 8) contributes more to the on-scalp signal than anticipated. In future recordings, more accurate methods for positioning and orienting on-scalp sensor arrays relative to the head should be achieved [16]. We propose that assessing orientation and location may be done offline using the signal from HPI-coils. With multi-sensor arrays, it will be possible to investigate what on-scalp MEG can offer in terms of source localization and spatial resolution.
